# Myocardial amyloidosis following multiple myeloma in a 38-year-old female patient: A case report

**DOI:** 10.1515/med-2020-0125

**Published:** 2020-05-16

**Authors:** Qisi Zhang, Yingli Qiao, Dongmei Yan, Yuhui Deng, Mengyang Zhang, Poshi Xu

**Affiliations:** Department of Clinical Laboratory, Henan Provincial People’s Hospital, Department of Clinical Laboratory of Central China Fuwai Hospital, Central China Fuwai Hospital of Zhengzhou University, Zhengzhou, Henan, 450003, China; Department of Clinical Laboratory, Affiliated Yancheng Hospital, School of Medicine, Southeast University, Jiangsu, Yancheng, 224001, People’s Republic of China; Department of Pathology Laboratory, Henan Province People’s Hospital, People’s Hospital of Zhengzhou University, Zhengzhou, Henan 450000, China

**Keywords:** multiple myeloma, amyloidosis, young-aged woman, case report

## Abstract

Multiple myeloma (MM) is an immunoglobulin-producing tumor of plasma cells, which occurs commonly in the elderly. The incidence of myocardial amyloidosis with MM is extremely low and early clinical manifestations are nonspecific. The diversity of clinical manifestations and first episode symptoms often cause misdiagnosis in young patients with myocardial amyloidosis following MM. In this study, we analyzed the clinical data of a young woman with MM and impaired cardiac function combined with echocardiography, electrocardiography (ECG), laboratory data, cell Congo Red staining, and other manifestations to diagnose amyloidosis. Considering the rapid progression, short survival, and poor prognosis in most patients, a clear, definitive, and timely diagnosis is essential for the treatment of patients with MM complicated with myocardial amyloidosis.

## 1 Introduction

Multiple myeloma (MM) is an immunoglobulin-producing tumor of plasma cells, which is commonly seen in the elderly [1,2]. The diversity of clinical manifestations and first episode symptoms often cause misdiagnosis in young patients with MM. The probability of amyloidosis in patients with MM is about 5–10% [3]. There are often no characteristic clinical symptoms initially. Considering the rapid progression, short survival, and poor prognosis in most patients, a clear and timely diagnosis is essential for the treatment of patients with MM complicated with myocardial amyloidosis. In this paper, we report the case of a 38-year-old woman with MM and heart failure. The granular sparkling pattern of myocardium was found on echocardiography that prompted further evaluation.

## 2 Case

**Figure 1:**
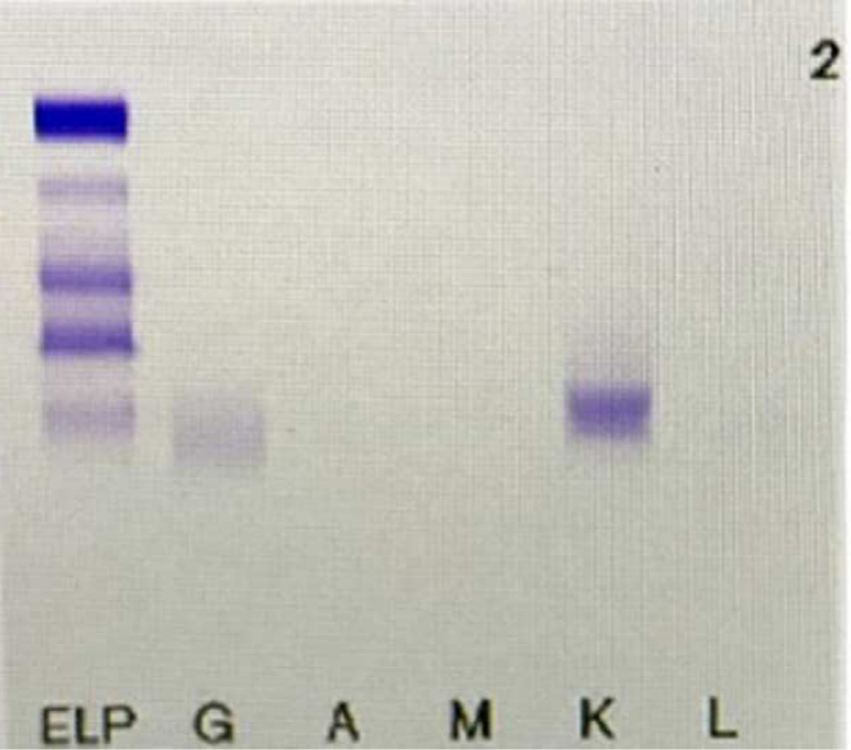
Serum immobilized electrophoresis (IFE) revealed positive result for κ-light chains.

**Figure 2:**
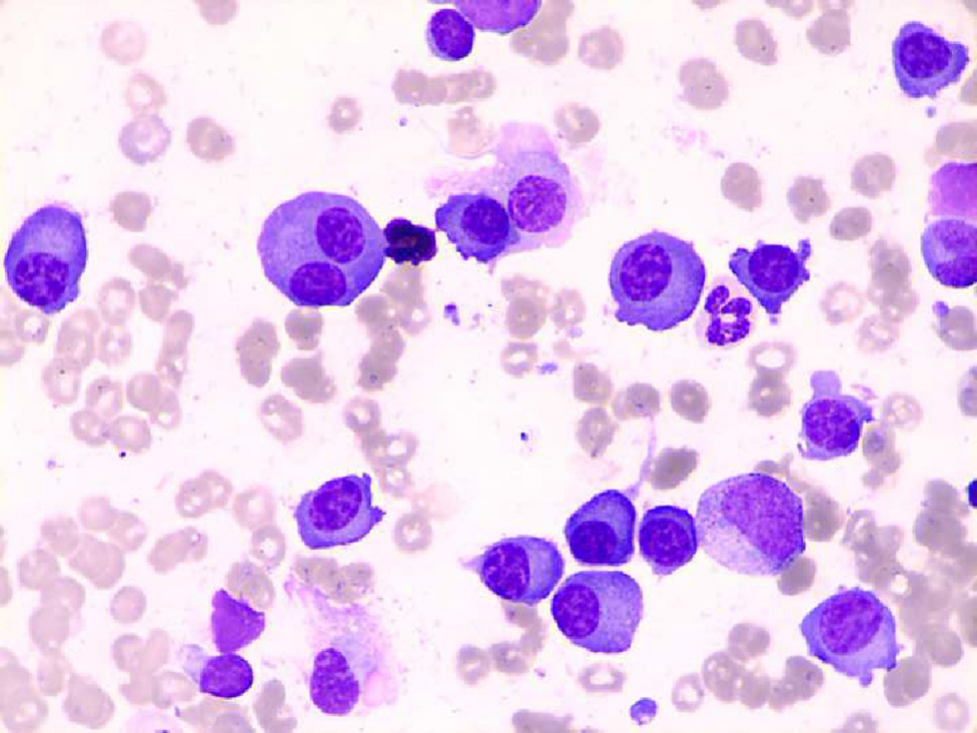
The bone marrow image showed an MM bone marrow change (Wright stain).

A 38-year-old woman was admitted to our hospital due to fatigue, bloating, edema of both lower extremities, and increased wheezing after activity in November 2018. Initial examination revealed creatinine elevation, left heart hypertrophy, and hypertension. She reported acute episodes of bloating, edema in the lower extremities, and wheezing during the previous 1 month; she denied history of any cardiovascular disease or family history of cardiovascular disease. Physical examination at admission showed pale skin and mucous membranes, enlarged liver (4–5 cm below the costal margin), edema of the lower limbs and varicose veins, and hypertension (blood pressure: 170/105 mm Hg). Laboratory results showed decreased total serum protein (52.6 g/L), serum albumin (33.9 g/L), and immunoglobulins (IgG, 2.05 g/L; IgA, 0.10 g/L; IgM, 0.01 g/L) as well as elevated 24-hour urinary total protein (2.93 g/24 h) (Table 1). Immuno fixation electrophoresis (IFE) revealed a positive result for κ-light chains (Figure 1). Color Doppler showed enhanced echogenicity of the kidneys. Computerized tomography showed bilateral pleural effusion; whole-body bone imaging or scan showed a mild increase in ingested radioactivity in the shoulder joint, sternum, and bilateral hip joints. For further evaluation, a bone marrow aspirate was performed. The bone marrow aspirate showed bone marrow changes typically seen in MM (Figure 2) and in combination with flow cytometry, plasma cells accounted for 58% of the total cell count. The results of fluorescence in situ hybridization showed that the 13q14 locus signal was deleted and the 14q32 locus gene rearrangement was positive. According to the National Comprehensive Cancer Network (NCCN) guidelines insights: MM (version 3.2016) [4], the condition of this female patient was diagnosed as MM.

**Table 1:** Laboratory results during hospitalization

Laboratory results (unit)	Jan 03, 2019	Mar 28, 2019	Apr 12, 2019	Jan 02, 2020
TP (g/L)	51.4	60.0	61.8	58.0
Serum albumin (g/L)	36.7	44.5	43.8	37.4
BUN (mmol/L)	21.4	22.0	11.1	14.2
Creatinine (µmmol/L)	239	221	205	418
eGFR (mL/min)	35.67	34.94	30.9	22.15
NT-proBNP (pg/mL)	17,871	59,940	86,208	2,81,168

TP = total serum protein; BUN = blood urea nitrogen; eGFR = estimation of glomerular filtration rate; NT-proBNP = N-terminal pro-B-type natriuretic peptide.

**Table 2:** Echocardiographic analysis results

Variables (unit)	Jan 03, 2019	Mar 28, 2019	Jan 02, 2020
LVEDV (mL)	124	93	93
LVESV (mL)	51	51	52
LVEF (%)	58.1	46.2	43.3
SV (mL)	72.1	43.0	40.4
LVIDD (mm)	51	42	45
LVIDS (mm)	35	34	35
FS (%)	30.9	23.0	21.6
Left atrial anteroposterior diameter (mm)	41	42	45
Left ventricle anteroposterior diameter (mm)	51	45	48
Right atrium transverse diameter (mm)	33	47	48
Right atrium longitudinal diameter (mm)	53	57	61
Right ventricular anteroposterior diameter (mm)	25	23	22
IVS (mm)	12	12	13
LVPW (mm)	12	12	13

LVEDV = left ventricular end-diastolic volume; LVESV = left ventricular end-systolic volume; LVEF = left ventricular ejection fraction; SV = Stroke volume; LVIDD = left ventricular end-diastolic diameter; LVIDS = left ventricular end-systolic diameter; FS = fractional shortening; IVS = interventricular septal thickness; LVPW = left ventricular posterior wall.

**Figure 3:**
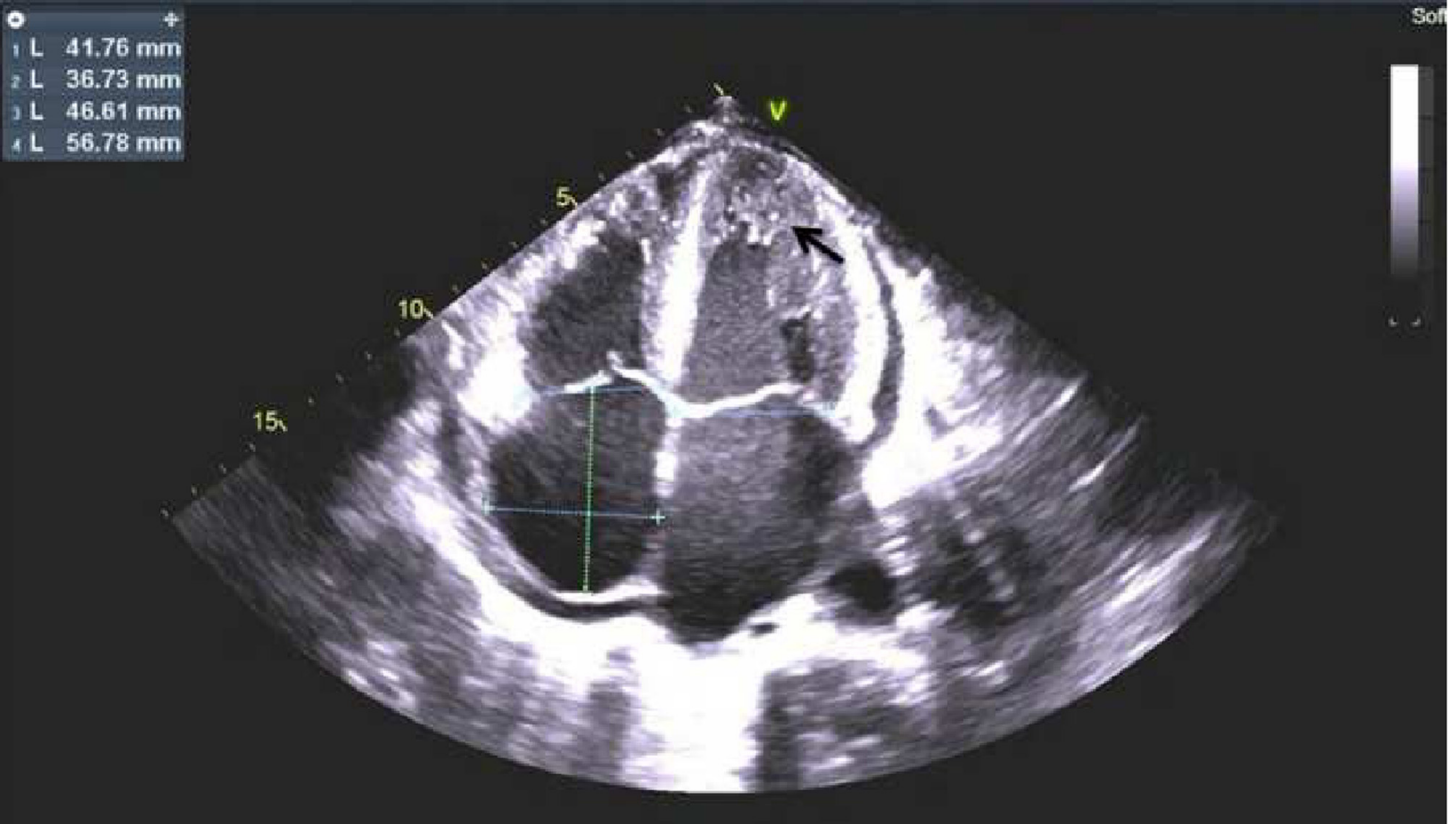
Echocardiography showing increased ventricular mass and granular sparkling pattern of myocardium (black arrows).

**Figure 4:**
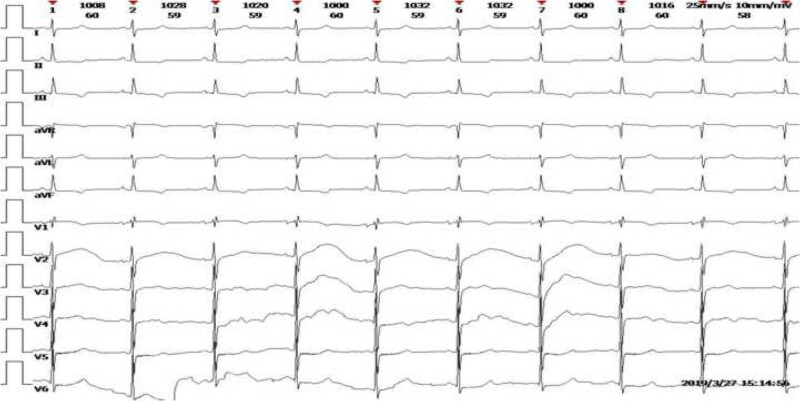
ECG showed ST-T abnormality after 3 months of admission.

**Figure 5:**
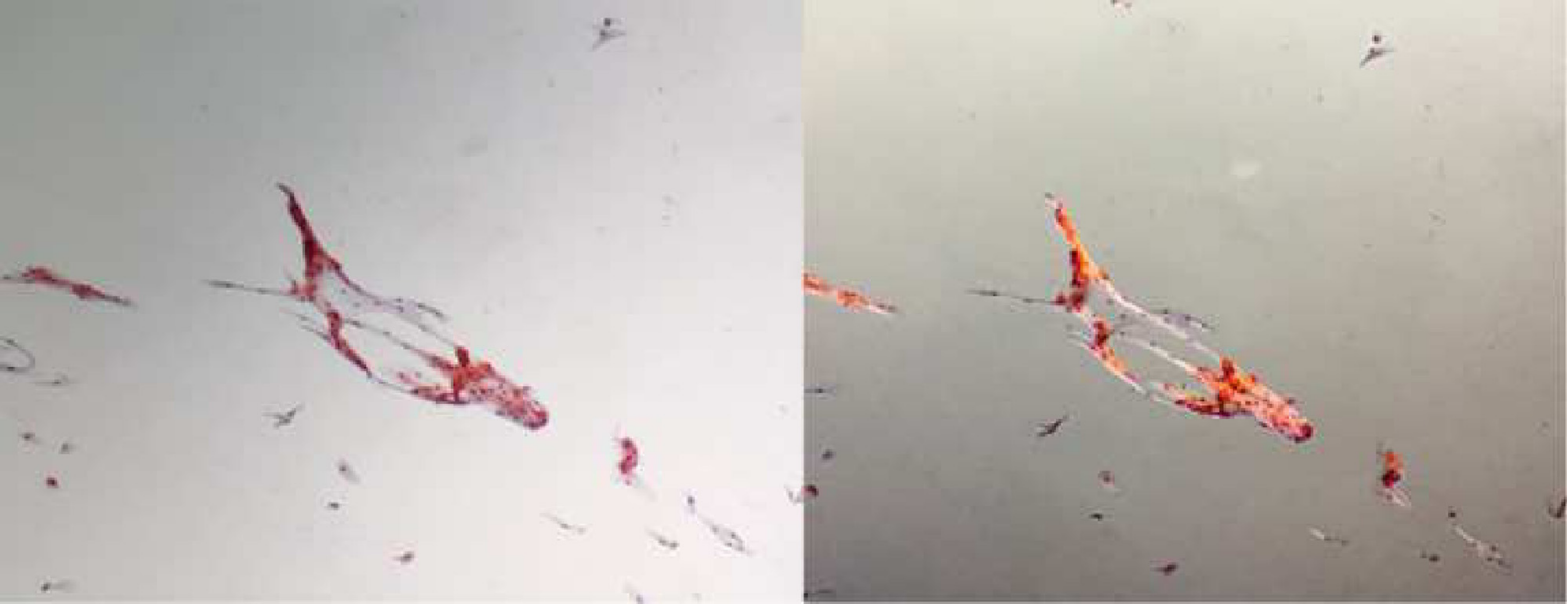
Oral mucosal exfoliated cells showing multiple deposits of amyloid, positive for Red Congo stain (left) and with yellow birefringence under polarized light (right).

During the hospital admission, the patient suffered from the rapid progress of wheezing, breathlessness in the sitting position, and evident chest tightness. Laboratory results revealed the following: N-terminal pro-B-type natriuretic peptide (NT-proBNP), 12,800 pg/mL; cardiac troponin T, 63.67 pg/mL; creatine kinase-MB, 9.02 ng/mL; and myoglobin, 97.68 ng/mL (Table 1). The initial echocardiography showed an enlarged heart, symmetrical thickening of the left ventricular wall, slightly enhanced echo, slightly reduced left ventricular systolic function (ejection fraction, 58%; fractional shortening, 30%), and minimal pericardial effusion. Mitral insufficiency, tricuspid insufficiency, and mild regurgitation were explored in the systolic phase. During hospitalization, echocardiography showed a further reduction in left ventricular systolic function and left ventricular diastolic function (Table 2) and granular sparkling pattern of the myocardium (Figure 3). One month after admission, electrocardiography (ECG) showed ST-T abnormalities as opposed to the initial normal ECG (Figure 4). In the next 3 months, the ECG still showed ST-T abnormalities and no low voltage performance. Cardiac magnetic resonance imaging (MRI) showed no subendocardial tissue enhancement by gadolinium. The combination of the New York Heart Association class III heart failure and history of MM was highly suggestive of a diagnosis of myocardial amyloidosis. Oral mucosal exfoliated cells showed positive staining for Congo Red stain (Figure 5). Furthermore, indentations on the tongue could be seen as the patient had macroglossia.


**Informed consent**: Informed consent has been obtained from patient included in this study.

## 3 Treatments

The patient was started on specific treatment with 40 mg of dexamethasone alone for 4 days. Immunosuppressants were replaced with a combination of intravenous (iv) bortezomib, 1.3 mg/m^2^ on days 1, 4, 8, and 11; thalidomide, 100 mg continuously; and dexamethasone 40 mg on days 1, 2, 8, 9, 15, 16, 22, and 23 for four 28-day cycles [5]. Diuretics are the mainstay of supportive care in cardiac amyloidosis. Furosemide (20 mg orally, once a day) and spironolactone (20 mg orally, once a day) in combination with hydrochlorothiazide (25 mg orally, three times a day) were used to alleviate cardiac preload and edema [6]. Torasemide (20 mg iv, once a day) was given instead of furosemide when the patient developed oliguria.

After four courses of treatment, plasma cells accounted for 71% of cell count on flow cytometry with the patient showing no remission. Progression of heart failure and renal failure was evident during the follow-up visit.

## 4 Discussion

The clinical manifestations and the initial symptoms of MM are diverse. MM usually occurs in people aged 65–74 years. The median age of onset is 69 years, and the ratio of male to female patients is about 1.58:1 [7]. As per statistics, only 3.2% of the patients are younger than 44 years, and patients aged 35 to 44 years account for 2.5% of the total cases [7]. Our patient was a 38-year-old young woman with renal and cardiac dysfunction as the initial manifestations. Abnormal blood cells and osteolytic symptoms were not evident. According to the diagnostic criteria of NCCN guidelines insights: MM [4], the patient was diagnosed as MM. However, this diagnosis can be easily missed because of the typical characteristics of susceptible populations. Therefore, young patients with MM should be carefully and comprehensively evaluated to arrive at the correct diagnosis.

The abnormal folding of light chains, rich in β leaves, due to changes in the secondary or tertiary structure of monoclonal immunoglobulins can form amyloid fibrils, which can lead to amyloidosis after extracellular deposition [8]. The probability of amyloidosis in patients with MM is about 10–15%. As with MM, amyloidosis was universally present in the elderly (median age, 64 years), and 99% were 40 years or older except one in a study comprising 474 patients [9]. Reports in recent years show the same results: 37 (60.7%) male, age 60 ± 11 years (*n* = 61) [10], 94 (74.0%) male, mean age, 61 years (*n* = 127) [11]. Amyloid deposits can occur in various organs, including heart, kidney, liver, and peripheral nervous system. Kidney involvement is the most common among the abovementioned organs and tissues with clinical cardiac involvement being the second most common presenting feature. According to a 2017 study, the mean age of patients with cardiac amyloidosis was 60 years (interquartile range, 54–68 years); 28 (59.6%) were men [10], which is analogous to the previous report (68 ± 10 years) [12]. Cardiac amyloidosis often has no characteristic clinical symptoms initially, but eventually heart failure ensues. For the Chinese population, aged 18–44 years, the reported average age of patients with heart failure and male and female patients are 36.82 ± 7.17 years, 37.44 ± 5.65 years, and 34.9 ± 7.17 years, respectively. The most common causes are dilated cardiomyopathy (31.0%), ischemic heart disease (29.6%), hypertensive heart disease (14.1%), peripartum cardiomyopathy (5.2%), and congenital heart disease (4.7%). Among male patients, ischemic heart disease and dilated cardiomyopathy are the most common causes, which account for 33.8% and 32.5% cases, respectively. Unlike male patients, dilated cardiomyopathy (26.4%) and peripartum cardiomyopathy (20.8%) are the most common causes among female patients [13]. Thus, a young female patient with myocardial amyloidosis following MM is rare and should be diagnosed cautiously.

ECG, echocardiography, and cardiac MRI are also important proofs for the diagnosis of myocardial amyloidosis. Studies have shown that the most characteristic ECG manifestation in patients with myocardial amyloidosis is the low-voltage pattern [14]. Typical echocardiography is characterized by thickening of the left ventricular wall with a granular sparkling appearance in the absence of hypertension and a limited or diffuse tissue enhancement of the heart by gadolinium on MRI. The combination of the characteristic findings of these three tests should be considered to make a diagnosis of cardiac amyloidosis. A study describing the clinical characteristics of eight patients with cardiac amyloidosis caused by MM reported that seven cases out of eight (87.5%) showed low limb lead voltage, six (75.0%) cases had poor precordial R-wave progression or pseudo-necrotic Q wave, and three (37.5%) cases presented with ST-T abnormalities [15]. This report showed different ECG characteristics in patients with myocardial amyloidosis. Low-voltage QRS complex also reportedly occurs in approximately 45–60% of the patients with AL amyloidosis; however, the absence of low-voltage QRS complex does not exclude the diagnosis of AL amyloidosis [11,16]. Echocardiography of this patient showed an enlarged heart, symmetrical thickening of the left ventricular wall, and left ventricular systolic dysfunction. There was no typical granular echo enhancement in the early stages of admission, which was seen only 4 months later. MRI did not show obvious enhancement by gadolinium. The initial ECG after admission was normal, but ST-T abnormalities appeared after 1 month, accompanied by increasing levels of NT-proBNP, combined with the rapid development of heart failure; however, there was no family history of heart disease; common heart diseases causing left heart failure were ruled out and the presence of cardiomyopathy was suggested. The patient had no symptoms of infection or bleeding, and there were no typical changes on MRI and echocardiography. This may be related to better cardiovascular functions, and the absence of complications, such as diabetes.

The pathological biopsy is the only means to confirm myocardial amyloidosis. Myocardial amyloidosis can be confirmed by combining positive results on ECG, echocardiography, and MRI with biopsy tissue Congo Red staining, but negative results of Congo Red staining do not rule out myocardial amyloidosis. However, because of poor cardiac function in patients with myocardial amyloidosis, blind examinations such as that of kidney tissue, abdominal adipose tissue, and intestinal tissue are often used as substitutes for myocardial biopsy. The positivity rates of different tissue specimens stained with Congo Red were reported as follows: abdominal fat, 50–80%; bone marrow, 56%; rectum, 75%; kidney, 94%; carpal ligament, 82%; liver, 97%; small intestine, 83%; skin, 90%; sural nerve, 86%; and heart, 100% [9,17]. The positivity rate of oral mucosa Congo Red staining in patients with primary amyloidosis has not been reported so far. However, the buccal mucosa was the most common site of amyloid deposition in the oral cavity, followed by the tongue, palate, gingiva, and floor of the mouth [18]. Systemic involvement is common: 90% of the patients with systemic amyloidosis will develop deposits in the head, neck, or respiratory tract [19]. About 25% of the patients with amyloidosis will develop macroglossia, the tongue becoming enlarged and firm, often with peripheral indentations from the teeth [20,21]. Recently, a study pointed out that among the 13 confirmed patients, 7 (54%) patients had clinical manifestations of oral amyloidosis (mainly macroglossia, indentations on the lateral border of the tongue, hematoma/ecchymosis), and in 5 of these 7 patients, the presence of amyloid deposition was confirmed by Congo Red staining of the diseased oral tissue [22]. The patient had indentations on the tongue and suffered from macroglossia. Because of the patient’s wishes, an invasive biopsy could not be taken. Therefore, we used a swab to scrape the oral mucosa for Congo Red staining. The Congo Red staining showed a yellow birefringence with apple-green birefringence under polarized light. This phenomenon is related to microscopy optics. If the optics are not perfect, which is almost inevitable on most microscopes, a mixture of colors is seen, typically blue-green and yellow-green, which can be called green and yellow, or even definite blue and yellow, without any green at all [23]. Combining the clinical and other features with the aforementioned literature reports, we suspected the presence of amyloidosis in the mouth and tongue. Considering that the patient had shown signs of multiple organ involvement (renal failure, heart failure, and macroglossia), the positive results of oral mucosa Congo Red stain confirmed systemic amyloidosis indirectly. Myocardial amyloidosis is more commonly seen than oral amyloidosis for the reason that the heart is the second most common site in amyloidosis. Combined with the typical echocardiography changes in myocardial amyloidosis, the positive result of oral mucosa Congo Red stain can indicate a diagnosis of highly suspicious for myocardial amyloidosis.

## 5 Conclusions

Myocardial amyloidosis should be considered in patients with MM presenting without subendocardial tissue enhancement by gadolinium on MRI and low voltage on ECG when Congo Red staining of biopsy tissue is positive; echocardiography shows the granular sparkling pattern of the myocardium and ECG shows ST-T abnormalities.
